# Acute Coronary Syndrome in Acute Myeloid Leukemia with Maturation Accompanying Megakaryocytic Differentiation

**DOI:** 10.1155/2020/8886298

**Published:** 2020-09-18

**Authors:** Kentaro Odani, Junya Abe, Yoshiaki Tsuyuki, Soshi Yanagita, Kazuya Shiogama, Mitsuhiro Tachibana, Yutaka Tsutsumi

**Affiliations:** ^1^Department of General Medicine, Shimada Municipal Hospital, Shimada, Shizuoka, Japan; ^2^Department of Diagnostic Pathology, Shimada Municipal Hospital, Shimada, Shizuoka, Japan; ^3^Department of Cardiology, Shimada Municipal Hospital, Shimada, Shizuoka, Japan; ^4^Department of Hematology, Shimada Municipal Hospital, Shimada, Shizuoka, Japan; ^5^Division of Morphology and Cell Function, Faculty of Medical Technology, Fujita Health University School of Health Sciences, Toyoake, Aichi, Japan; ^6^Diagnostic Pathology Clinic, Pathos Tsutsumi, Nagoya, Aichi, Japan

## Abstract

An autopsy case (85-year-old Japanese male) of myeloperoxidase- (MPO-) positive acute myeloid leukemia with maturation (M1) accompanying megakaryocytic differentiation is presented. The patient manifested acute coronary syndrome. Even after emergent percutaneous coronary intervention, his performance status remained poor, so no chemotherapy against leukemia was given. The final white blood cell count reached 291,700/*μ*L, and the platelet count was elevated to 510,000/*μ*L. No cytogenetic studies were performed. He died at the 25^th^ day of hospitalization. Autopsy revealed marked leukemic infiltration to the endocardium and subendocardial myocardium. Subendocardial myonecrosis was surrounded or replaced by the leukemic blasts, and neither granulation tissue reaction nor fibrosis was observed. In the cardiovascular lumen, lard-like blood clots were formed and microscopically consisted of leukemic blasts and platelets (leukemic thrombi). Infiltration of leukemic blasts was seen in the body cavities and systemic organs including the lung. The MPO-positive blasts lacked azurophilic granules and expressed the stem cell markers, CD34 and CD117 (c-kit). No features of myelofibrosis were seen in the 100% cellular marrow. In the endocardium, liver, lymph nodes, and bone marrow, megakaryocytic cells (CD42b/CD61+, MPO-) were distributed, while the small-sized blastic cells in the blood and tissues predominantly expressed MPO. The blasts lacked expression of CD42b/CD61. Megakaryocytic differentiation might be stimulated by certain tissue factors. AML accompanying megakaryocytic differentiation in certain tissues and organs should be distinguished from acute megakaryoblastic leukemia. The mechanisms provoking acute coronary syndrome in acute myeloid leukemia are discussed.

## 1. Introduction

Acute coronary syndrome has been reported as the initial manifestation of acute leukemia: leukemia cells may provoke coronary obstruction [1–5]. After chemotherapy, mycotic embolism should also be considered a cause of acute coronary syndrome in leukemia patients [6].

We report herein an autopsy case of MPO-positive AML with maturation (M1) of the aged accompanying megakaryocytic differentiation in certain tissues and organs. Because of poor performance status, no chemotherapy was given, nor cytogenetic studies performed. Of note, the patient manifested acute coronary syndrome, likely due to leukemia cells-induced intravascular thrombosis and direct invasion of leukemia cells to the heart muscle.

## 2. Case Presentation

An 85-year-old Japanese male with a past history of brain stroke and chronic obstructive pulmonary disease was transferred to Shimada Municipal Hospital, Shimada, Shizuoka, Japan, because of acute chest discomfort. Electrocardiogram indicated acute coronary syndrome. Emergent percutaneous coronary intervention was carried out. The catheter occluded the left main trunk, resulting in cardiopulmonary arrest. Cardiopulmonary resuscitation rescued spontaneous circulation. A covered stent was then grafted to the narrowed portion of the left coronary artery. A peripheral blood examination revealed an abnormally high level of white blood cells (93,900/*μ*L) with 83.5% myeloblasts positive for myeloperoxidase (MPO) activity. Hemoglobin count was 5.1 g/dL, and the platelet count was 510,000/*μ*L. Since his performance status was judged insufficient for chemotherapy, no treatment was given against AML. The patient tentatively recovered to having a meal, but he died of respiratory failure 25 days after hospitalization. The final white blood cell count reached 291,700/*μ*L. Neither fluorescence-activated cell sorting (FACS) analysis, karyotyping, nor precise cytogenetic studies were performed.

### 2.1. Pathological Findings

Autopsy clarified lard-like (white colored and elastic) blood clots in the cardiac and aortic lumen, as well as in the pericardial and pleural spaces. The blood clot in the cardiac lumen weighed 80 g. The bilateral pleural cavities were filled with lard-like material, and the left cavity contained 1,100 mL effusion (right side: unmeasurable). Microscopically, the clot consisted of blastic cells, intermingled with numbers of platelets. The leukemic blasts infiltrated the bone marrow (100% cellularity without myelofibrosis), liver (1,000 g), spleen (200 g), and lymph nodes (maximal size: 15 mm). The blasts also involved the serosal membranes, heart (410 g), lung (left 330 g, right 420 g), kidney (left 250 g, right 190 g), adrenal glands (left 10 g, right 5 g), and testis, as well as, though mildly, the pancreas, vermiform appendix, prostate, and seminal vesicles. Dense leukemic infiltration into the lung parenchyma and pleural cavity, associated with massive effusions and secondary organizing/aspiration pneumonia, was regarded as the direct cause of death. There was no microscopic evidence of extramedullary hematopoiesis: no erythroblastic islands were identified in the extramedullary organs and tissues. The brain was not examined. The final autopsy diagnoses are summarized in Table 1.

**Figure 1:**
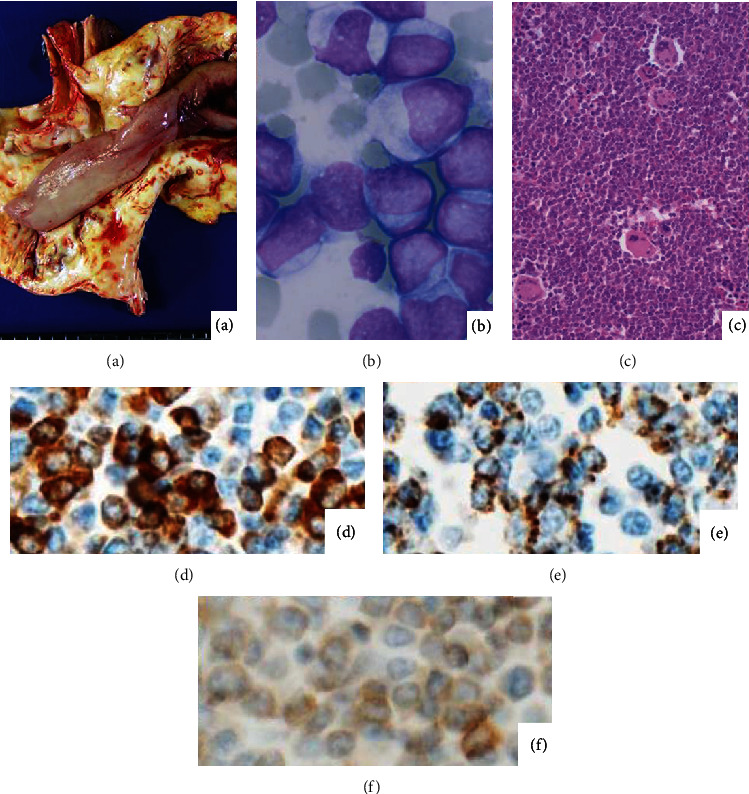
Features of leukemia cells ((a) gross appearance of lard-like blood clot in the aorta; (b) Giemsa-stained cytology of leukemia cells; (c) hematoxylin and eosin- (H&E-) stained histology of the bone marrow; immunostaining for MPO (d), CD34 (e), and CD117 (f)). The coagulated leukemia cells form white-colored elastic clots in the vascular space. The leukemic blasts in pleural effusion show high nuclear/cytoplasmic ratio and lack azurophilic granules. The bone marrow reveals 100% cellularity occasionally with megakaryocytic differentiation. The blasts in the bone marrow are immunoreactive for MPO, CD34, and CD117 (c-kit).

**Figure 2:**
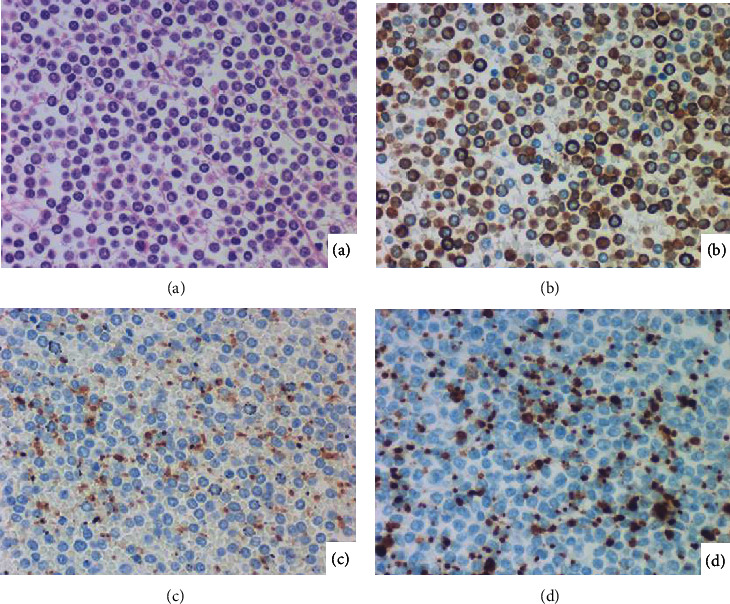
Histopathological appearance of blood clot in the atrium ((a) H&E; immunostaining for MPO (b), CD42b (c), and CD61 (d)). Most of the small-sized blastic cells in the blood express MPO. CD42b and CD61 label platelets among the blasts, but the blasts are devoid of expression of the megakaryocytic markers.

The blastic cells in May-Giemsa-stained preparations possessed no azurophilic granules in a scanty basophilic cytoplasm. No megakaryoblastic cells were observed. The marker study performed using formalin-fixed paraffin-embedded sections disclosed expression of MPO and the stem cell markers such as CD34 and CD117 (c-kit). CD3, CD10, CD20, CD79a, and terminal deoxynucleotidyl transferase were negative.  Figure 1 demonstrates lard-like leukemic thrombosis in the aorta, leukemic blasts in the pericardial space, and bone marrow histology.  Figure 2 illustrates a high-powered microscopic view of blood clot in the atrial lumen. The small-sized blasts were diffusely positive for MPO, while immunostaining for CD42b and CD61 decorated platelets. The blasts lacked expression of CD42b and CD61.

**Figure 3:**
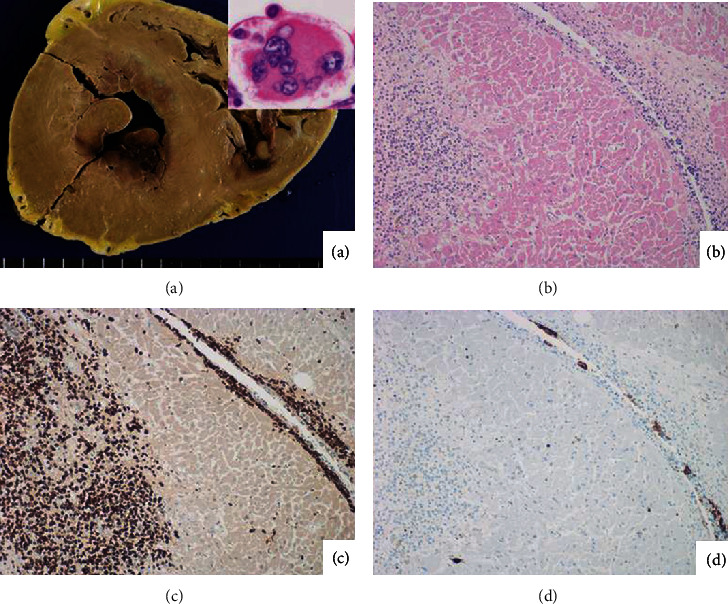
The heart involved by leukemic cells ((a) cut surface of the heart after formalin fixation; (b) H&E, inset: high-powered view; (c) MPO; (d) CD61). Grossly, the subendocardial myocardium circumferentially appears dark-colored. Microscopically, the MPO-positive leukemic blasts invade both the myocardium and endocardium. CD61-positive megakaryocytic cells are distributed in the endocardium. The inset demonstrates high-powered H&E morphology of a subendocardial located megakaryocyte. The infiltrating blasts replace the cardiomyocytes. Neither granulation tissue reaction nor fibrosis is seen in the involved myometrium.

**Figure 4:**
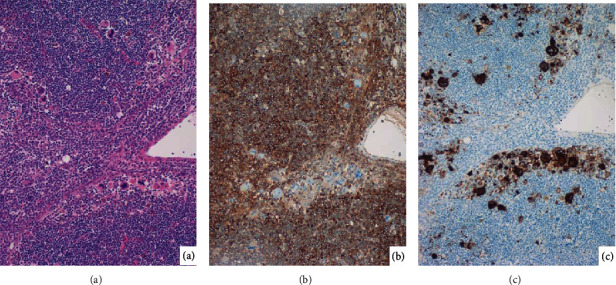
Lymph node histology ((a) H&E, (b) MPO, and (c) CD61). The nodal parenchyma is diffusely infiltrated by MPO-positive leukemic blasts. CD61-positive megakaryocytic differentiation is observed mainly in the sinus. The small-sized blasts lack expression of CD61.

The horizontal cut surfaces of the heart grossly displayed subendocardial ischemia, and microscopic examination demonstrated dense infiltration of leukemic blasts among cardiac muscle cells. Cardiomyocytes were replaced by the leukemic blasts, or coagulation necrosis of the muscle cells was densely surrounded by the leukemic blasts. In spite of the clinical course for 25 days, the common histopathologic features of myocardial infarction, such as granulation tissue reaction and fibrosis, were hardly seen in the myocardium, and coronary arterial atherosclerosis was mild. The leukemic infiltration was also noted in the endocardium, where megakaryocytic differentiation was microscopically evident. Immunohistochemically, the small-sized monomorphic blasts in the myocardium and endocardium were MPO-positive and CD42b/CD61-negative, while MPO-negative megakaryocytic cells strongly expressed CD42b/CD61, as illustrated in Figure 3. Morphological differentiation toward CD42b/CD61-positive megakaryocytes was also observed in the bone marrow (see Figure 1), lymph nodes, and liver.  Figure 4 demonstrates diffuse infiltration of MPO-positive and CD61-negative blastic cells in the lymph node parenchyma and CD61-positive and MPO-negative megakaryocytic differentiation mainly in the sinus. The blastic cells expressing CD42b/CD61 were not observed.

## 3. Discussion

We presented here an autopsy case of acute myeloid leukemia (AML) with maturation accompanying megakaryocytic differentiation in certain tissues and organs. First, differential diagnosis of acute megakaryoblastic leukemia (AMKL) is needed. AMKL belongs to acute myeloid leukemia, not otherwise specified, acute megakaryoblastic leukemia according to the World Health Organization classification 2017, or M7 according to the French-American-British (FAB) system. AMKL is defined as an AML with >20% blasts, of which >50% belong to the megakaryocyte lineage [7–10]. AMKL comprises 4–15% of AML in children [11, 12], but in adults, AMKL occurs in <1% of AML patients [13]. Leukemic blasts in AMKL are commonly characterized by the expression of CD31, CD41, CD42b, CD43, and CD61 without MPO reactivity, and myelofibrosis is often associated in the bone marrow [7–13]. AMKL with MPO activity has also been described [14–16]. Tallman et al. reviewed 20 adult cases morphologically diagnosed as AMKL to find two MPO-positive lesions [7]. Deficient expression of CD11a has been reported as another marker of AMKL [17]. Expression of the stem cell markers is inconsistent in AMKL [10, 18]. Histopathologic features of MPO-negative AMKL reveal marked pleomorphism of neoplastic cells, ranging from myeloblast-like small round cells to large cells with an abundant cytoplasm, in the fibrotic bone marrow tissue [19]. When multilineage myeloblastic proliferation is found and the percentage of blasts is low, the diagnosis of acute panmyelosis with myelofibrosis is made [18].

We described herein an adult case of AML with strong MPO immunoreactivity and the expression of stem cell markers (CD34 and CD117). Myelofibrosis was absent in the leukemic marrow. Of note, there was distinctive differentiation toward CD42b/CD61-positive megakaryocytes in the bone marrow, lymph nodes, liver, and endocardium. Supposedly, such megakaryocytic differentiation was induced by certain tissue factors. Reports say that megakaryocytic differentiation is accelerated by thrombopoietin (c-myeloproliferative leukemia (c-Mpl) ligand) [20], protease-activated receptor 3 (PAR3) [21], serum response factor (SRF)/myocardin-related transcription factor A (MRTFA) complex [22], and megakaryoblastic leukemia 1 (MKL1), an activator of SRF, promoted by Ras homolog family member A- (RhoA-) induced actin polymerization [23]. It has been reported that phenotypic conversion from AML, M0 to AMKL occurred at the time of relapse [24, 25].

Tissue-dependent differentiation toward megakaryocytes may be related to a hidden potential of differentiation in the immature blastic cells of AML. Theoretically, there might be a separate population of dysplastic megakaryocytes with no actual labeling of CD42b/CD61 on the morphologic blasts. Or a minor population of blasts might express CD42b/CD61. It is difficult for us to get the direct morphological proof for such suspicion. During the hematopoiesis, a series of committed progenitors produce all kinds of blood cells, and megakaryocytes are derived through the multipotent progenitor, common myeloid progenitor, or megakaryocyte-erythroid progenitor [26]. Hematopoietic stem cells expressing the megakaryocyte-specific integrin CD41 have a myeloid bias and are activated by a megakaryocyte-specific, zinc finger-type transcription factor, GATA-binding protein-1 (GATA1) [27]. It is supposed that the leukemic blasts expressing stem cell markers in the present case derived from the common myeloid progenitor committed to myeloblastic and megakaryoblastic differentiation, and the tissue factors induced megakaryocytic differentiation within the tissue and organ.

The association of thrombocytosis in the peripheral blood was related to the megakaryocytic differentiation, a unique feature of our case. Acute megakaryoblastic leukemia accompanying thrombocytosis has been reported [28, 29]. In the present case, the leukemic small-sized blasts seen in the blood and infiltrating to the tissues lacked expression of CD42b/CD61. The fact indicated the importance of autopsy for characterizing the nature of leukemic blasts. The diagnosis in the present case should be AML with maturation (FAB M1) accompanying megakaryocytic differentiation, instead of AMKL.

The formation of lard-like blood clot consisting of leukemic blasts and platelets in the cardiovascular lumen and pleural cavities was quite characteristic of the present case. The platelet count in the peripheral blood was elevated to 510,000/*μ*L. The patient suffered from acute coronary syndrome as the initial manifestation of acute leukemia, as has been reported previously [1–5]. The proposed mechanisms for acute leukemia-associated acute coronary syndrome include: (a) leukemic thrombosis or leukostasis in the coronary artery, (b) leukemic infiltration into the myocardium, (c) hypercoagulative status secondary to leukemia, and (d) antileukemic therapy causing cardiovascular adverse effects [1, 4]. After chemotherapy, mycotic embolism is a possible cause of coronary obstruction in leukemia patients [6]. In the present case, leukemic thrombosis (leukostasis) in the coronary artery seemed to be closely related to the initial symptom of acute coronary syndrome, since the formation of lard-like white colored clots is solely composed of leukemic blasts and platelets. Thrombocytosis-associated hypercoagulability likely contributed to the thrombotic process. In addition, dense infiltration of MPO-positive and CD42b/CD61-negative leukemic blasts into the subendocardial myocardium with loss or coagulation necrosis of cardiomyocytes was also noticeable. The leukemic blasts probably infiltrated the heart parenchyma during the 25-day clinical course. All of these processes might act as causative factors for the acute coronary symptoms and signs. Leukemia should be considered one of the nonatherosclerotic etiologies of acute coronary syndrome.

## Figures and Tables

**Table 1:** The final anatomical diagnosis (85-year-old male).

(i) Acute myeloid leukemia (FAB M1) with maturation accompanying megakaryocytic differentiation
(a) No treatment given (clinical course: 25 days)
(b) Marker expression: CD34+, CD117+ (c-kit), MPO+, CD42b-, CD61-, CD10-, TdT-, CD3-, CD20-, and CD79a-
(c) Infiltration of leukemic blasts
(1) Leukemic bone marrow (100%)
(2) Liver (1,000 g)
(3) Spleen (200 g)
(4) Lymph nodes (max. 15 mm)
(5) Serosal membranes: (ascites 70 mL; pleura effusion left 1,100 mL, right: unmeasurable; pericardial effusion 80 mL)
(6) Lung (multifocal, severe; left 330 g, right 420 g)
(7) Subendocardial myometrium and endocardium (heart weight 410 g)
(8) Lard-like blood clot in the heart, aorta, and pleural and pericardial cavities
(9) Leukemic infiltration to the kidney (left 250 g, right 190 g), adrenal glands (left 10 g, right 5 g), testis > pancreas, vermiform appendix, prostate, and seminal vesicles
(d) Megakaryocytic differentiation seen in the bone marrow, lymph nodes, liver, and endocardium
(ii) Secondary pneumonia (severe): organizing pneumonia and aspiration pneumonia
Direct cause of death: respiratory failure due to leukemic infiltration to the lung and pleura
